# Supplementary material for: Serum creatinine and estimated glomerular filtration rate (eGFR) in early pregnancy and changes during the pregnancy
Source: PLOS Glob Public Health. 2023 Jan 26;3(1):e0000443. doi: 10.1371/journal.pgph.0000443 (PMC10021427; doi:10.1371/journal.pgph.0000443)
Supplement: S1 Text — (DOCX) [file pgph.0000443.s002.docx]

**Variable definitions:**

**Serial:** serial number, **Maternal_Ethnicity:** ethnicity of the pregnant women, **Gravidity: gravidity, Age_years:** age of the pregnant women in completed years**, Period_of_Gestation: the** period of gestation at the time of sample collection**, SCr_mol/L:** serum creatinine**, eGFR_EPI,** serum creatinine-based estimated glomerular filtration rate.
