# Supplementary material for: Serum creatinine and estimated glomerular filtration rate (eGFR) in early pregnancy and changes during the pregnancy
Source: PLOS Glob Public Health. 2023 Jan 26;3(1):e0000443. doi: 10.1371/journal.pgph.0000443 (PMC10021427; doi:10.1371/journal.pgph.0000443)
Supplement: Attachment — Submitted filename: PLoSGHP_reply.docx [file pgph.0000443.s003.docx]

**Editors comment**
In the online submission form, you indicated that "The datasets generated during and/or analyzed during the current study are available from the corresponding author on reasonable request." require all data underlying the findings described in their manuscript to be freely available to other researchers, either 1. In a public repository, 2. Within the manuscript itself, or 3. Uploaded as supplementary information.

The dataset is uploaded as supplementary information.

**Comments to the Author**

**Reviewer #1: These data are very useful for producing a local reference range for serum creatinine in pregnancy and the creatinine data provided should be made available for future meta-analyses. However there are some limitations which restrict generalisability to a global readership:**
**1. Reported creatinine concentrations are limited to 1st and 2nd trimesters, yet most creatinine concentrations measurements in pregnancy are taken in the third trimester, where AKI is most prevalent due to peripartum events.**

We agree with the comment that AKI is common in the third trimester. Many reported studies are on that trimester. It’s the same reason we wanted to do this study to report the Scr value in the first trimester. To report the baseline values to identify AKI in the third trimester in future studies (if there is an association)

**2. It is not clear whether the serum creatinine method is an isotope dilution mass spectometry traceable creatinine assay**

Thanks and we added this to the methodology isotope dilution mass spectrometry traceable creatinine assay

**3. eGFR is reported as a measure of kidney function in pregnancy even though it is acknowledged that it consistently underestimates function. Existing published data on the association of eGFR with adverse pregnancy outcomes utilises eGFR as a biomarker, rather than an absolute measure of kidney function. This is distinct from the establishment of a reference interval of eGFR as a marker of kidney function, which is flawed.**

We agree with the comment that eGFR is not a marker of kidney function. We have made some changes (including the title) to clarify that we are not declaring it as a marker of kidney function. Even if it is an underestimation, population normality data is always useful in epidemiological analysis (probably not for individual patient care).

**4. Up to one-third of CKD presents for the first time in pregnancy. Although a known diagnosis of CKD and the presence of hypertension at the time of the study are exclusion criteria, an opportunity was missed to exclude haematuria and proteinuria, which may have helped to make the data more robust.**

Thank you very much for the excellent suggestion. We have excluded haematuria and proteinuria as suggested and reanalysed the dataset to make it more robust. The entire results section is edited with this reanalysis.

**5. The value of the data to other centres, especially given the local prevalence of CKDu, is not clear. The data are certainly smaller than that reported by others using a single assay eg JAMA. 2019;321(2):205-207. doi:10.1001/jama.2018.17948, n=243,534.**

We appreciate the fact that the data set is “small” compared to the JAMA article. We explained the difference in the article itself. However, ethnicity has a major effect on renal function. The JAMA article is from US data and may not be applicable to the south Asian context. For South Asian women, the “normality data” are not reported so far. In addition, the JAMA article is not using “healthy” population data. The particular dataset is from routinely available data, usually biased with “unhealthy” visits. Our one is a population sample after excluding those with probable kidney diseases. We have discussed these issues in the article.

**Reviewer #2:**

This is a prospective study of serum Cr values in early pregnancy and throughout gestation, up to 30 weeks. The authors define 'normal' ranges for pregnancy in the healthiest population they could identify and compare it to a similar population. This is an important paper and adds solid, clear data to a field with a lot of ambiguity. It is helpful to have this data in the South Asian population, specifically, as well.

I have a few small points:
**1. I am a bit confused about women who were included. In the methods, it states 'only women who made it 24 weeks were included', but then there seems to be a separate group studied for the repeated measures analysis. In Table 3, I do not really understand what the N column is referring to - is this just the number of women who had their first Cr drawn at that point in gestation but that whole group definitely had a check at 24 weeks? Some more explanation is needed, I think on this point as to the number of blood draws each woman had and what the different groupings mean.**

We have edited the methods section for clarity. In table 4 title, we have stated that this is 500 women with the “paired sample”, which means all these 500 women had first trimester and second-trimester blood samples checked. We have edited the title as well.

**2. There are a lot of statistical methods described in the results, but not elaborated on in the methods. Would make sure to describe the tests and why they were chosen and used in the methods, as well.**

Actually, one-way, two-way and repeated measure ANOVA were the three statistical methods used. All others mentioned components are part of the ANOVA analysis. We have added the description in the methods

**3. Is the effect of age on eGFR just due to the fact that age is in the CKD-EPI equation in a non-linear format? Age is an exponent, so I would anticipate that there would be non-linear effects of age on eGFR in every circumstance. If there is more to it than this, which there very well could be, would explain in the discussion what the meaning of the interaction testing, etc. you did in the results and how people should be thinking about age when determining what values are outside the normal range in pregnancy, if at all.**

After the reanalysis with all suggested exclusion criteria, age was not associated with eGFR and this discussion was removed.
